# *Strongyloidiasis stercoralis* coinfection is associated with altered iron status biomarkers in tuberculous lymphadenitis

**DOI:** 10.3389/fimmu.2022.999614

**Published:** 2022-10-20

**Authors:** Gokul Raj Kathamuthu, Anuradha Rajamanickam, Rathinam Sridhar, Dhanaraj Baskaran, Subash Babu

**Affiliations:** ^1^ National Institutes of Health-NIRT-International Center for Excellence in Research, Chennai, India; ^2^ Indian Council of Medical Research-National Institute for Research in Tuberculosis (ICMR-NIRT), Chennai, India; ^3^ Government Stanley Medical Hospital, Chennai, India; ^4^ Laboratory of Parasitic Diseases, National Institute of Allergy and Infectious Diseases, National Institutes of Health, Bethesda, MD, United States

**Keywords:** tbl, *Strongyloides stercolaris*, iron status biomarkers, hematology, elisa

## Abstract

Soil-transmitted helminth [mainly *Strongyloidiasis stercoralis* (Ss)] and tuberculous lymphadenitis (TBL) coinfection in humans is a significant public health problem. We have previously shown that TBL+Ss+ coinfection significantly alters diverse cytokine, matrix metalloproteinase, and tissue inhibitors of metalloproteinase profiles. However, no data is available to understand the influence of Ss coinfection in TBL disease with respect to iron status biomarkers. Hence, we have studied the effect of Ss coinfection on the circulating levels of iron status (ferritin, transferrin [TF], apotransferrin [ApoT], hepcidin, hemopexin) biomarkers in TBL disease. Our results show that TBL+Ss+ and/or TBL+Ss- individuals are associated with significantly altered biochemical and hematological (red blood cell (RBC) counts, hemoglobin (Hb), hematocrit (HCT), mean corpuscular volume (MCV), mean corpuscular hemoglobin (MCH) were decreased, and platelets were increased) parameters compared to TBL-Ss+ individuals. Our results also show that TBL+Ss+ coinfection is associated with diminished circulating levels of ferritin, ApoT, hepcidin, and hemopexin compared to TBL+Ss- individuals. TBL+Ss+ and TBL+Ss- groups are associated with altered iron status biomarkers (decreased ferritin [TBL+Ss+ alone] and increased TF, ApoT, hepcidin and hemopexin [TBL+Ss- alone]) compared to TBL-Ss+ group. The heat map expression profile and principal component analysis (PCA) analysis of iron status biomarkers were significantly altered in TBL+Ss+ compared to TBL+Ss- and/or TBL-Ss+ individuals. A significant correlation (positive/negative) was obtained among the biochemical and hematological parameters (white blood cells (WBC)/ferritin, TF, and hepcidin, mean corpuscular hemoglobin concentration (MCHC)/ferritin and hemopexin) with iron status biomarkers. Finally, receiver operating characteristic (ROC) analysis revealed that hemopexin was significantly associated with greater specificity and sensitivity in discriminating TBL+Ss+ and TBL+Ss- coinfected individuals. Thus, our data conclude that Ss coinfection is associated with altered iron status biomarkers indicating that coinfection might alter the host-*Mtb* interface and could influence the disease pathogenesis.

## Introduction

Pulmonary tuberculosis (PTB) is caused by *Mycobacterium tuberculosis* (*Mtb*) and remains one of the primary causes of (1.5 million) death reported worldwide (1). Extrapulmonary tuberculosis (EPTB), especially tuberculous lymphadenitis (TBL) is the most frequent manifestation with the cervical region mostly affected (2). Globally, 2 billion people are affected by helminth infections and both *Mtb* and helminth-associated infections share a vast degree of geographical overlap (3, 4). Worldwide, 27% of the people are infected with *Mtb*, while 24% are infected with helminth infections (3, 4). One among the helminth infections is *Strongyloides stercoralis* (Ss) a soil-transmitted helminth that affects around 100 million individuals (3). Thus, understanding the influence of helminth coinfection with TB disease is crucial because the immunological responses to helminths could influence the ability to control *Mtb* disease and treatment outcomes (5). The interaction between TBL and Ss is less studied in terms of immune-mediated outcomes and only two such studies have been reported previously (6, 7). The function of host-mediated immune protection against TB disease requires innate and adaptive immunity but diverse other components that might contribute to the establishment and/or reactivation of TB disease are not known (8, 9).

One such molecule is iron and both host and mycobacterial growth need iron which primarily acts as a cofactor and is also essential for the successful activation of the immune system (10). Too much free iron is also lethal to the cell and in turn acts as a resource for invading microorganisms (11). In humans, *Mtb* often establishes a complicated system of procuring, metabolizing and storing necessary iron (deficit and surplus). Although the host strives to hamper the iron accessibility to the mycobacteria, still *Mtb* thrives in utilizing the iron molecules such as lactoferrin, ferritin, and transferrin (Tf) (12). Therefore, TB disease-associated iron metabolism is gaining bigger attention. Diverse studies have revealed that iron status biomarkers could assist in the clinical diagnosis of *Mtb* disease (13–16) and most notably poor delivery of iron acts as the hallmark of TB-related anemia (17, 18). Earlier studies have reported the involvement of iron metabolism as risk factors for the development of TB disease among household contacts in TB disease with diabetes mellitus (DM) or HIV comorbidities and as potential markers of TB treatment failure (19–23).

However, to our knowledge, no studies have shown the iron status biomarker systemic levels in TBL disease upon Ss coinfection. Thus, we have examined the plasma levels of ferritin, transferrin [TF], hepcidin, hemopexin and apotransferrin [ApoT] iron status biomarkers in TBL disease with Ss coinfection (TBL+Ss+), TBL disease without Ss coinfection and TBL negative with Ss coinfection groups. Our results indicate that the iron status biomarker levels are significantly altered among the TBL+Ss+ group compared to TBL+Ss- and TBL-Ss+ group. Overall, we conclude that Ss coinfection might be associated with altered iron status biomarkers indicating that coinfection might alter the host-*Mtb* interface and could influence the TBL disease pathogenesis.

## Methods

### Ethics

The ICMR-National Institute for Research in Tuberculosis (NIRT), Institutional ethics committee (IEC 2010007) has approved the protocol and written consent was taken from all patients involved in the study.

### Study subjects

Totally, 132 patients were enrolled in the present study. Among them, 44 individuals were positive for both TBL disease and Ss infection (hereafter indicated as TBL+Ss+, n=44), 44 individuals were positive for TBL disease and negative for Ss infection (hereafter indicated as TBL+Ss-, n=44) and 44 individuals were negative for TBL disease and positive for Ss infection (hereafter indicated as TBL-Ss+, n=44). The study demographics and hematological data were shown in **Table 1**. A convenience sample was recruited as part of a natural study protocol, as previously described (7). The positivity of TBL disease was determined based on histopathological or bacteriological examination (GeneXpert or culture) from the dissected cervical lymph nodes using culture [0 (no colonies)/1+ (20-100 colonies)/2+(>100 colonies)] on Lowenstein-Jensen solid media. We used recombinant NIE (31 kDa) antigen to detect the existence of IgG antibodies by ELISA to diagnose the Ss infection positivity (24, 25). At baseline, all the study participants were naïve to both anti-tuberculosis and anthelmintic treatment. The study subjects were not affected by human immune deficiency virus (HIV), disseminated strongyloidiasis, or filarial infection.

**Table 1:** Study demographics and hematological parameters of the individuals.

Parameters	TBL+Ss+	TBL+Ss-	TBL-Ss+	P-Value*
Patients enrolled (n)	44	44	44	–
Gender (Male/Female)	12/32	14/30	25/19	–
Age in years	23 (18-53)	25 (19-59)	40.2 (19-64)	–
Culture grades (0/1+/2+)	7/30/7	15/28/1	–	0.028^a^
Lymphocytes (%)	1.96 (0.88-3.49)	1.97 (0.84-3.51)	2.75 (1.08-4.01)	P<0.0001
Neutrophils (μL)	4313.2 (2251.6-7077)	3712 (2042.6-6418)	3907 (1658.8-8798.4)	P=0.0472
Monocytes (%)	419.3 (102.9-889.2)	552.1 (213-1331.2)	524.9 (218.5-1482)	P=0.0017
Eosinophils (%)	3.25 (0.6-8.7)	2.83 (0.8-13.2)	10.1 (2.6-29.2)	P<0.0001
Basophils (%)	0.99 (0.3-6.7)	0.94 (0.2-2.6)	1.14 (0.1-6.6)	NS

a Mann-Whitney U test and *Kruskal-Walli’s test was used to calculate the significance; NS, non-significant.

### Plasma collection and storage

The plasma was separated from the heparin-coated (BD Vacutainer^®^) whole blood tubes after being centrifuged for 10 minutes at 1,460 Relative Centrifugal Force (RCF) or G-Force and was precisely transferred to new sterile screw-cap tubes (Sarstedt). They were further stored at -80°C for future experimental use.

### Plasma ELISA

Iron biomarkers such as ferritin (Orgentec Diagnostika), apotransferrin (apoT), hepcidin (USCN Life Science Inc), transferrin (TF), and hemopexin (genway) were measured by ELISA. The lowermost detection limit of ferritin is 15 ng/mL; transferrin, 9.375 ng/mL; apotransferrin, 0.156 ng/mL; hemopexin, 6.25 ng/mL and hepcidin, 62.5 pg/mL.

### Hematology

The hematological (lymphocytes, neutrophils, monocytes, basophils and eosinophils) and biochemical (red blood cell (RBC), white blood cell (WBC), platelets, hemoglobin (Hb), hematocrit (HCT), mean corpuscular volume (MCV), mean corpuscular hemoglobin (MCH), mean corpuscular hemoglobin concentration (MCHC) and red blood cell distribution width (RDW) data among the TBL+Ss+ and TBL+Ss- individuals were measured using an AcT5 Diff hematology analyzer (Beckman Coulter).

### Statistical analysis

The iron status biomarkers were analyzed using GraphPad Prism (version 9.0, GraphPad Software, Inc., San Diego, CA, USA). We performed a non-parametric Kruskal-Walli’s test (Geometric means [GM] and central tendency) to find the statistical differences among study subjects. The significant difference between the culture grades was evaluated using the Mann-Whitney U test. Heatmap expression profile and correlation analysis were performed between TBL+Ss+, TBL+Ss-, and TBL-Ss+ groups using GraphPad Prism. JMP^®^ Statistical Software (version 13.0) was used to calculate the principal component analysis (PCA) for TBL+Ss+ and TBL+Ss- infected groups. ROC curves were also performed between TBL+Ss+ and TBL+Ss- groups to measure the power of each iron status biomarker. Linear trend analyses (Kolmogorov-Smirnov (distance)) was used to measure the significance between culture grades and iron biomarkers.

## Results

### Demographics

The study population data comprising demographics and hematological parameters are described in **Table 1**. The study groups were not significantly different in terms of age or gender. However, we found that the culture grades were significantly different between the two TBL-infected groups. In addition, lymphocytes, monocytes, neutrophils, and eosinophils were found to be statistically significantly different among the three (TBL+Ss+, TBL+Ss-, TBL-Ss+) groups of infected individuals.

### TBL+Ss+ coinfection associated with diminished hematological (except platelets) parameters

**Figure 1:**
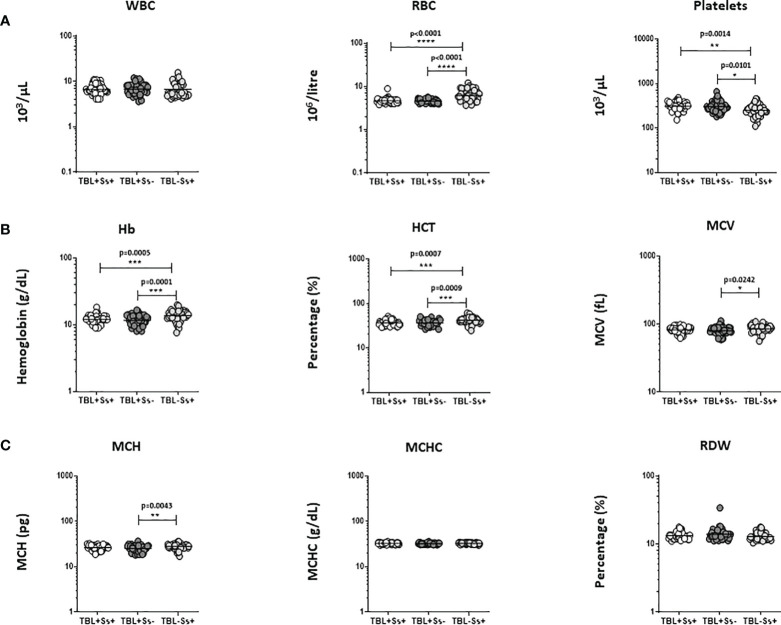
Altered hematological parameters of the population. We have analyzed different hematological **(A)** WBC, RBC, platelets **(B)** Hb, HCT, MCV **(C)** MCH, MCHC, RDW) parameters in TBL+Ss+ (n=44), TBL+Ss- (n=44) and TBL-Ss+ (n=44) groups using the AcT5 Diff hematology analyzer (Beckman Coulter). We used Kruskal-Walli’s test with Dunn’s multiple comparisons to measure the statistical P values. The significance of p values was represented as '*' p<0.05, '**' p<0.01, '***' p<0.001, '****' p<0.0001.

We assessed the hematological ([A] WBC, RBC, platelets, [B] Hb, HCT, MCV, [C] MCH, MCHC and RDW) parameters between the study groups **(** **Figures 1A–C** **)**. **Figure 1** displays that TBL+Ss+ and TBL+Ss- coinfection showed significantly reduced RBC, Hb and HCT levels and increased platelet counts when compared to TBL-Ss+ group. TBL+Ss- group was associated with decreased MCV and MCH levels in comparison with the TBL-Ss+ group alone. There was no difference in the WBC, MCHC, and RDW levels between the study groups. Thus, TBL+Ss+ and/or TBL+Ss- coinfection is associated with altered hematological parameters.

### Systemic iron status biomarkers were altered in TBL+Ss+ coinfection

**Figure 2:**
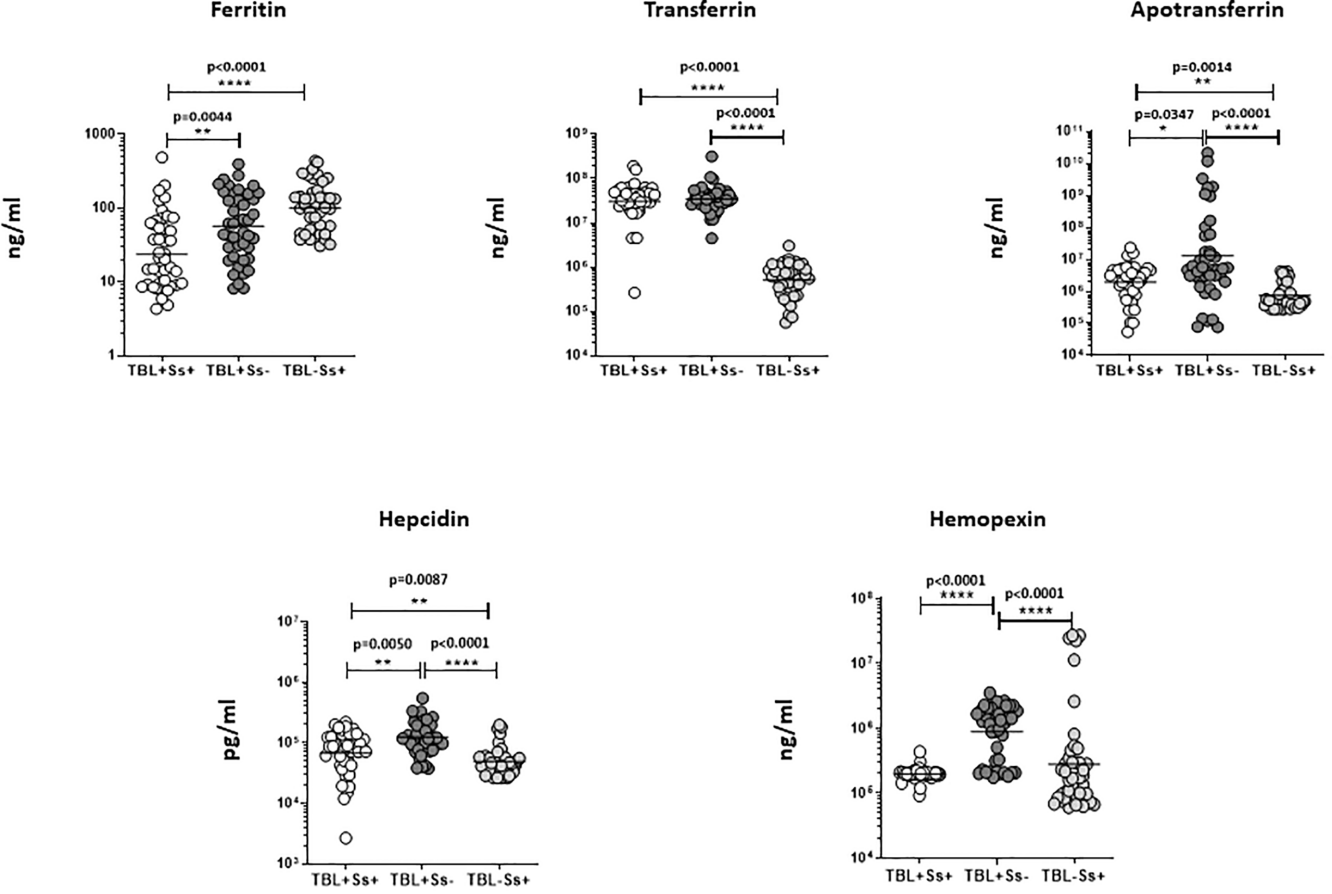
Iron status biomarker plasma levels in TBL+ (Ss+, Ss-) and TBL-Ss+ groups. We have measured the iron status (ferritin, transferrin [TF], apotransferrin [ApoT], hepcidin, hemopexin) biomarker systemic levels in TBL+ (Ss+, n=44), (Ss-, n=44) and TBL-Ss+ (n=44) groups by ELISA. We showed the figures using scatter plots with an individual circle denoting a single participant. We used Kruskal-Wallis Dunn’s multiple comparisons test to measure the statistical P values. The significance of p values was represented as '*' p<0.05, '**' p<0.01, '****' p<0.0001.

We have examined iron status (ferritin, transferrin, apotransferrin (apoT), hepcidin, and hemopexin) biomarker systemic levels in TBL+Ss+, TBL+Ss-, and TBL-Ss+ groups **(** **Figure 2** **)**. The ferritin (GM of 23.58 ng/ml in TBL+Ss+ versus 57.26 ng/ml in TBL+Ss-, and 101.8 ng/ml in TBL-Ss+) systemic levels were significantly decreased in TBL+Ss+ than TBL+Ss- and TBL-Ss+ groups. However, transferrin (GM of 30688319 ng/ml in TBL+Ss+ and 34663199 ng/ml in TBL+Ss- versus 512836 ng/ml in TBL-Ss+), apoT (GM of 1988954 ng/ml in TBL+Ss+ and 12971073 ng/ml in TBL+Ss- versus 740983 ng/ml in TBL-Ss+) and hepcidin (GM of 67837 pg/ml in TBL+Ss+ and 123426 pg/ml in TBL+Ss- versus 49234 pg/ml in TBL-Ss+) systemic levels were increased significantly in TBL+Ss+ and TBL+Ss- group in comparison with TBL-Ss+ group. Finally, the apoT (GM of 1988954 ng/ml in TBL+Ss+ versus 12971073 ng/ml in TBL+Ss-), hepcidin (GM of 67837 pg/ml in TBL+Ss+ versus 123426 pg/ml in TBL+Ss-) and hemopexin (GM of 196312 ng/ml in TBL+Ss+ versus 882775 ng/ml in TBL+Ss-) systemic levels were significantly decreased in TBL+Ss+ than TBL+Ss- group **(** **Figure 2** **)**. Hence, iron status biomarkers are significantly altered in TBL+Ss+ coinfection when compared to the other groups.

### Heatmap, PCA, and correlation analysis

**Figure 3:**
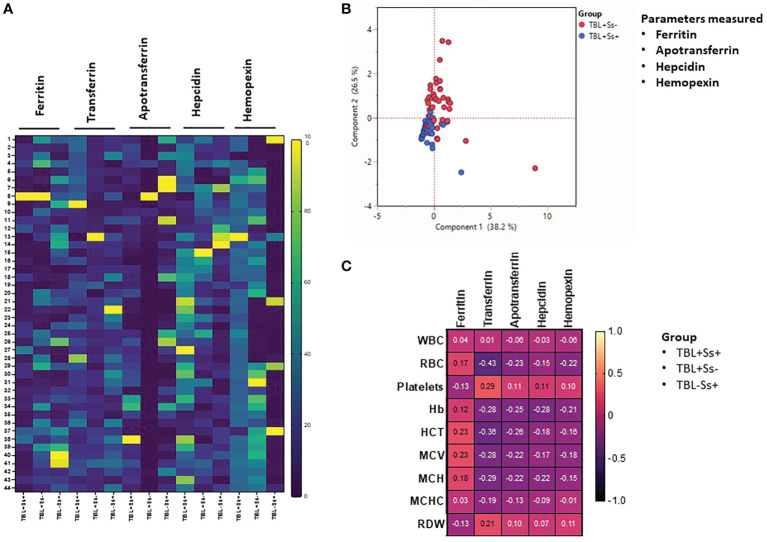
Heatmap, principal component analysis (PCA) and correlation analysis of iron status biomarkers. **(A)** Heatmap analysis of iron status (ferritin, TF, ApoT, hepcidin, hemopexin) biomarkers was performed between TBL+Ss+ (n=44), TBL+Ss- (n=44) and TBL-Ss+ (n=44) individuals. **(B)** PCA analysis of iron status (ferritin, transferrin [TF], apotransferrin [ApoT], hepcidin, hemopexin) biomarkers was carried out between TBL+Ss+ (n=44, blue circle) and TBL+Ss- (n=44, red circle) to examine the cluster pattern between component 1 versus component 2. **(C)** Correlation ability of iron status (ferritin, transferrin [TF], apotransferrin [ApoT], hepcidin, hemopexin) biomarkers and hematological (WBC, RBC, platelets, Hb, HCT, MCV, MCH, MCHC, RDW) parameters between TBL+Ss+ (n=44), TBL+Ss- (n=44) and TBL-Ss+ (n=44) individuals.

We studied the heatmap expression of iron status biomarkers after normalizing their plasma levels among different study groups **(** **Figure 3** **)**. TBL+Ss+ individuals had significantly decreased ferritin expression compared to TBL+Ss- and TBL-Ss+. Hepcidin and hemopexin expression was significantly increased in TBL+Ss+ compared to TBL+Ss- and/or TBL-Ss+. TBL+Ss- group was associated with significantly decreased TF and ApoT expression compared to TBL+Ss+ and TBL-Ss+ **(** **Figure 3A** **)**. We also generated the PCA models and assessed the influence of iron status (ferritin, apotransferrin, hepcidin, and hemopexin) biomarkers between TBL+Ss+ and TBL+Ss-. We show iron status biomarkers form distinct clustering among PCA [component 1 (38.2%) and component 2 (26.5%)] analysis between TBL+Ss+ and TBL+Ss- individuals **(** **Figure 3B** **)**. Finally, we performed the correlation analysis of iron biomarkers with different hematological (WBC, RBC, platelets, Hb, HCT, MCV, MCH, MCHC, RDW) parameters of TBL+Ss+, TBL+Ss-, and TBL-Ss+ **(** **Figure 3C** **)**. We show that WBC versus ferritin, TF (positve correlation) and ApoT, hepcidin (negative correlation) was signifcanly correlated; whereas, MCHC versus ferritin (positive correlation), and hemopexin (negative correlation) were significantly correlated among the study population. Thus, iron status biomarkers were significantly expressed, discriminated against, and correlated among different study groups.

### ROC analysis of iron status biomarkers

**Figure 4:**
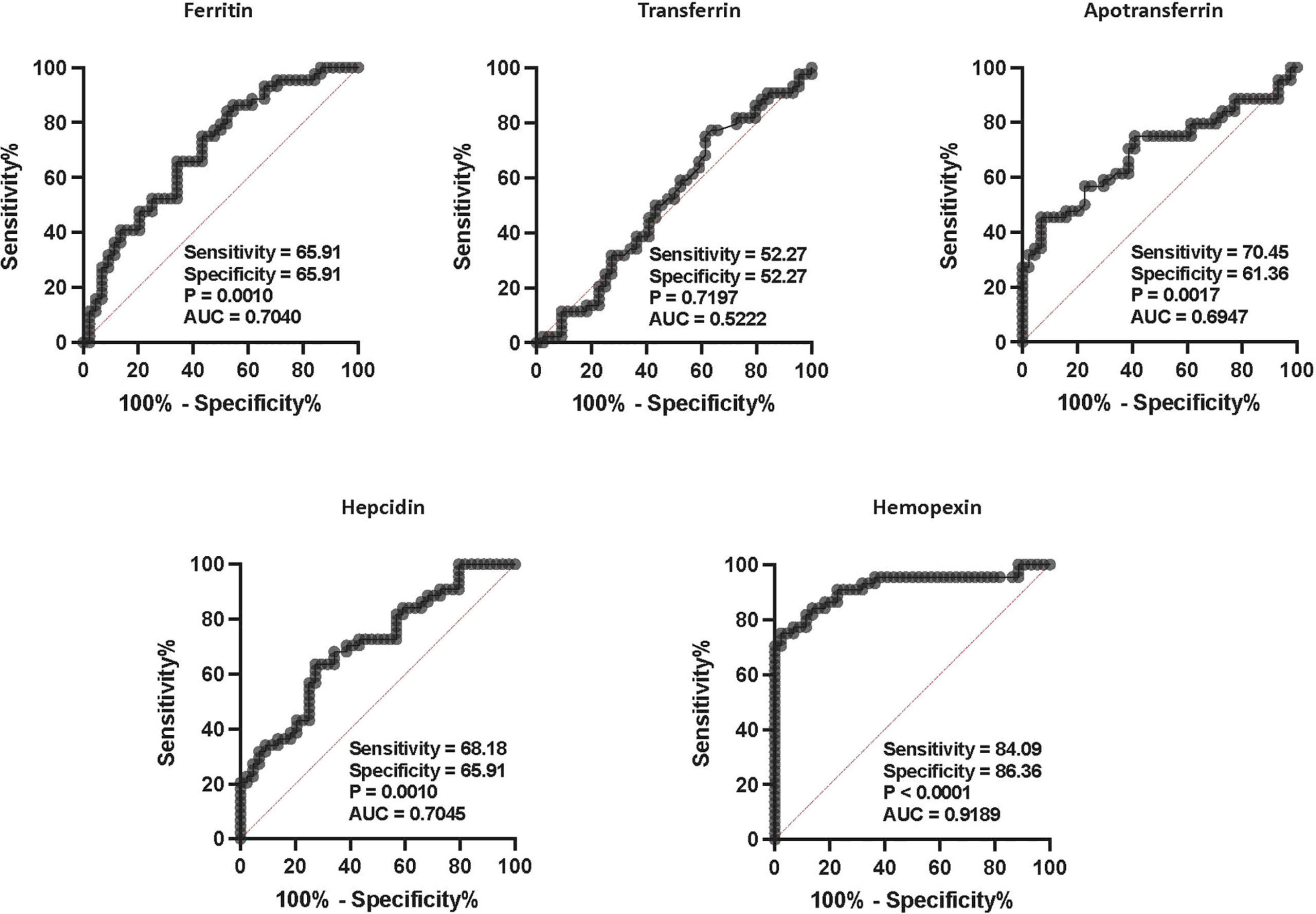
ROC analysis of iron status biomarkers. ROC analysis of iron status (ferritin, TF, ApoT, hepcidin, hemopexin) biomarkers in TBL+ (Ss+, n=44 and Ss-, n=44) groups was performed to evaluate the sensitivity, specificity, and area under the curve.

We calculated the effect of iron status biomarkers in discriminating/differentiating TBL+Ss+ from TBL+Ss- and TBL-Ss+. Thus, we have used ferritin, transferrin, apotransferrin, hepcidin, and hemopexin plasma levels to calculate the ROC analysis between the three groups. As shown in **Figure 4**, hemopexin (sensitivity-84.09, specificity-86.36, AUC- 0.9189, P< 0.0001) showed higher significant discriminatory power in distinguishing TBL+Ss+ in comparison with TBL+Ss-. However, the other iron status biomarkers (ferritin [sensitivity-65.91, specificity-65.91, AUC- 0.7040, P= 0.0010], transferrin [sensitivity-52.27, specificity-52.27, AUC- 0.5222, P= 0.7197], apotransferrin [sensitivity-70.45, specificity-61.36, AUC- 0.6947, P= 0.0017], hepcidin [sensitivity-68.18, specificity-65.91, AUC- 0.7045, P= 0.0010]) did not display significant discriminatory power to discriminate TBL+Ss+ from TBL+Ss- individuals **(** **Figure 4** **).** Thus, hemopexin reveals the discriminatory potential to aid as a biomarker to differentiate TBL+Ss+ from TBL+Ss- individuals.

### Linear trend analysis of iron status biomarkers

**Figure 5:**
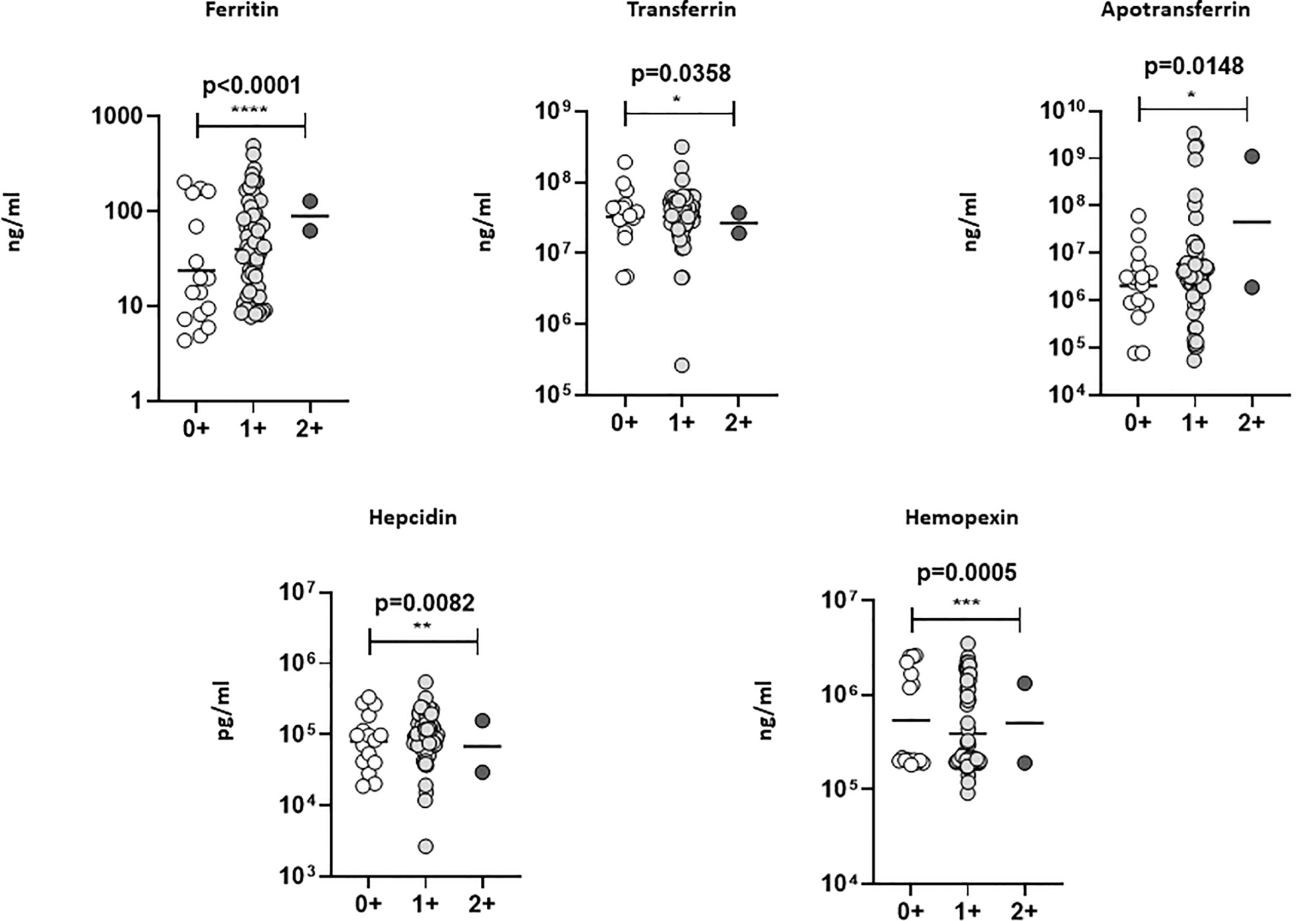
Linear (trend) regression analysis of iron status biomarkers. The association between plasma levels of iron status biomarkers was correlated with lymph node culture grades (TBL+ (Ss+, n=44 and Ss-, n=44) individuals) using linear trend analysis (one-way ANOVA). The results were shown as scatter plots with each circle denoting a single individual. The significance of p values was represented as '*' p<0.05, '**' p<0.01, '***' p<0.001, '****' p<0.0001.

To understand the clinical association of iron status biomarkers with TBL and Ss coinfection, we performed the regression analysis with culture (0+, 1+, 2+) grades for TBL+Ss+ and TBL+Ss- groups and compared them with plasma levels of iron status biomarkers. Our results describe that iron status biomarkers were significantly associated with culture grades. However, ferritin and apotransferrin levels were significantly increased and transferrin, hepcidin and hemopexin levels were decreased as the disease severity elevates **(** **Figure 5** **)**. Thus, iron status biomarkers are significantly associated with disease severity.

## Discussion

Iron is necessary for all organisms to regulate diverse biological process and *Mtb* inhabit the phagosome that has restricted contact with iron (26, 27). Iron is required for the host interaction as well as for *Mtb* metabolism which is either depleted intracellularly from the cytoplasm (28) or through the synthesis of iron-chelating particles called siderophores and macromolecules (13, 29–33). The relative risk factor of iron biomarkers in disease progression and pathogenesis of TB and TB-HIV-coinfection has been reported earlier (19, 23). However, no previous study has shown iron status biomarker levels in tuberculous lymphadenitis (TBL) disease with *S. stercoralis* (Ss) coinfection. Hence, our study provides a detailed investigation of iron status biomarkers in TBL+Ss+ coinfection. To our knowledge, for the first time, we are showing the temporal association of iron status (ferritin, transferrin [TF], apotransferrin [apoT], hepcidin, hemopexin) biomarkers in TBL-Ss+ coinfection using a human cohort. We show TBL+Ss+ individuals associated with altered iron status biomarkers than TBL+Ss- and TBL-Ss+ individuals.

Ferritin acts on iron homeostasis upon binding and sequestering the iron intracellularly and increased levels are naturally linked with both severe and persistent inflammatory milieu (34). Ferritin behaves as an acute phase response marker and increased ferritin levels are correlated to a higher risk of death in TB and TB-DM comorbidities (22, 35–37). In contrast, our results suggest that ferritin levels were lower and found to be statistically different in TBL+Ss+ and TBL+Ss- when compared to TBL-Ss+ individuals. Reduced levels of iron are linked with weakened immune function (38, 39) and greater susceptibility or development of TB disease. Deficit levels of iron might compromise cell-mediated immune responses through diminished stimulation of lymphocytes and macrophage bactericidal action (39). Iron deficiency could possibly alter the Th1/Th2 cytokine equilibrium and stimulate strong Th2 immunity which is known to be correlated with clinical TB disease (40, 41). Also, insufficient alimentary Fe, imperfect absorption to hookworm or other subordinate infections, and greater blood loss could decrease the storage of body iron (42).

Transferrin is another essential iron transport and iron-binding glycoprotein with the ability to regulate and release free iron levels into the tissues and erythroblasts (43). An increased level of transferrin-iron saturation in serum is associated with greater death and morbidity in TB disease. It is perhaps because extracellular mycobacteria that exist in a transferrin-affluent milieu exploit this pathway for instant iron access (13, 22). Transferrin and lactoferrin aid in the growth of both *Mtb* and *M. avium* which are known to acquire host proteins to the phagosome (32, 33, 44). Similarly, apoT acts as an endogenous immune modulator and has a biological function in the passage and delivery of iron among the different body parts (45). It has been previously elucidated that long-term ex-vivo treatment with apoT primes to down-regulation of the detrimental Th1 and Th17 autoimmune responses (46, 47). Our results demonstrate that both transferrin and apoT were higher in the TBL+ (Ss+ and Ss-) groups when compared with TBL-Ss+ group. In comparison with our study, previous data on PTB coinfection with diabetes mellitus or HIV disease is associated with reduced transferrin levels (21, 48). The reason for this observation is yet to be explored; however, increased levels might associate with enhanced inflammatory responses associated with TBL disease with or without coinfection. Also, the presence or absence of Ss coinfection did not alter the transferrin levels. One potential reason might be because *Mtb* but not *Ss* utilizes the host-cell transferrin iron acquisition pathway to scavenge iron [13].

Numerous studies have shown the role of hepcidin in host iron status among TB diseases (19, 49, 50). We reveal that increased hepcidin could possibly associate with anemia because iron availability for erythropoiesis is reduced. Hepcidin regulates the iron supply of the host and has impact on the result of infection, which depends upon the niche of the microorganism (51, 52). Synthesis and expression of hepcidin were increased during inflammation or surplus iron storage (53). It possesses antimicrobial function and emerges to have a significant role in innate immunity against TB disease (51, 54). In contrast to ferritin, hepcidin plasma levels were increased and found to be statistically significant in TBL+Ss+ and TBL+Ss- groups compared to the TBL-Ss+ group. Higher systemic levels of hepcidin might be associated with anemia of inflammation and could possibly reflect the detrimental effects of iron supplementation often noticed during infections (49). Previous studies on mouse models have revealed that hepcidin-stimulated iron capture could be favorable for intracellular microbes which include *Mtb* (55, 56).

Finally, we also studied the plasma levels of hemopexin in TBL+Ss+, TBL+Ss- and TBL-Ss+ groups. We reveal that reduced hemopexin levels were associated with TBL+Ss+ and TBL-Ss+ groups than TBL+Ss- coinfected group. Hemopexin potentially induces anti-inflammatory responses and suppresses the pro-inflammatory role in binding to free hemoglobin. It is also vital in the retrieval of heme-iron by binding to hemoglobin and modifying the receptor expression for heme-oxygenase, ferritin, and transferrin markers (57, 58). Thus, upon coinfection TBL individuals might not efficiently activate the necessary inflammatory responses. We also studied the heatmap analysis of iron biomarkers between the three groups of study individuals and showed they were differentially expressed among TBL+ (Ss+, Ss-) and TBL-Ss+ groups. PCA analysis of iron (ferritin, hepcidin, apoT and hemopexin) biomarkers significantly discriminated in TBL (Ss+, Ss-) groups. Iron status biomarkers were also significantly correlated with some of the hematological parameters. ROC analysis revealed that hemopexin was associated with higher sensitivity and specificity in discriminating between the study (TBL+Ss+ and TBL+Ss-) groups. In addition, iron status biomarkers were significantly associated with culture grades. In a clinical context, deficiency in the iron biomarkers along with coinfection might reduce anti-TB treatment efficacy or delay culture conversion and elevate mortality. We did not include healthy controls in our analysis due to the following reasons. Our main hypothesis was to understand how the coinfected individuals have different iron marker levels than a single infection or disease. Also, iron biomarkers were strongly connected with disease pathogenesis, and this infers that anemia might be a consequence of the disease state but not with a healthy state. Overall, strongyloidiasis coinfection plays a distinct function and might alter the host iron and/or bacterial iron accessibility as well as could perhaps augment the jeopardy of progression and pathogenesis in TBL disease. Thus, a better understanding of the helminth coinfection to discover pathways and crucial interventions associated with TBL disease is urgently needed.

## Data availability statement

The original contributions presented in the study are included in the article/supplementary material. Further inquiries can be directed to the corresponding author.

## Ethics statement

The ICMR-National Institute for Research in Tuberculosis (NIRT) and institutional ethics committee (IEC 2010007) has approved the protocol and written consent was taken from all patients involved in the study. The patients/participants provided their written informed consent to participate in this study.

## Author contributions

GK and SB conceived and designed the experiments. GK and AR performed the experiments. GK analyzed the data. DB, RS, and SB contributed materials/reagents/analysis tools. GK and SB wrote the paper. All authors contributed to the article and approved the submitted version.

## Funding

This research work was supported by the Division of Intramural Research, National Institute of Allergy and Infectious Diseases (NIAID), National Institute of Health.

## Acknowledgments

The authors thank V. Rajesh Kumar of NIH-ICER, the staff of the Department of Clinical Research, ICMR-NIRT, Government Stanley Hospital, Government General Hospital, and Government Kilpauk Medical Hospital, Chennai for valuable assistance in recruiting the patients for this study. This work was supported by the Division of Intramural Research, NIAID, NIH. The funders have no role in study design, data collection or analysis, data interpretation, or manuscript writing.

## Conflict of interest

The authors declare that the research was conducted in the absence of any commercial or financial relationships that could be construed as a potential conflict of interest.

## Publisher’s note

All claims expressed in this article are solely those of the authors and do not necessarily represent those of their affiliated organizations, or those of the publisher, the editors and the reviewers. Any product that may be evaluated in this article, or claim that may be made by its manufacturer, is not guaranteed or endorsed by the publisher.
